# Programmed cell death ligand 1 in correlation with *BRAF^V600E^* mutation, molecular characteristics and biological behavior in ameloblastoma

**DOI:** 10.3389/fimmu.2026.1762600

**Published:** 2026-06-16

**Authors:** Aijing Pan, Zhipeng Xie, Jianhui Zhan, Yifeng Zhao, Yue Du, Kai Zhang

**Affiliations:** Department of Oral and Maxillofacial Surgery, The First Affiliated Hospital of Bengbu Medical University, Bengbu, Anhui, China

**Keywords:** ameloblastoma, CD8+ T lymphocytes, immunotherapy, molecular targeted therapy, PD-L1

## Abstract

**Objective:**

To investigate the expression of Programmed cell death ligand 1 (PD-L1) in Ameloblastoma (AM) and its correlation with *BRAF^V600E^* mutation as well as the expression of Cytotoxic CD8^+^ T cells (CD8^+^ T) and Forkhead box protein 3 (FoxP3) in the immune microenvironment. And to preliminarily explore the potential association of PD-L1 with the biological behavior and Disease-free survival (DFS) of patients with AM, so as to provide a theoretical reference for clinical diagnosis and treatment.

**Methods:**

Fifty formalin-fixed paraffin-embedded (FFPE) specimens of AM were enrolled in this study. Immunohistochemistry (IHC) was performed to detect the expression of *BRAF^V600E^* mutant protein, CD8^+^ T cells, FoxP3^+^ T cells and PD-L1. Molecular testing for the *BRAF^V600E^* mutation was further conducted in 29 eligible AM specimens. PD-L1 expression was evaluated by IHC, and the combined positive score (CPS) ≥ 1 was defined as PD-L1 positivity. The chi-square test and Fisher’s exact test were used for bivariate statistical analysis. Survival analysis was performed using the Kaplan-Meier method with the log-rank test, and the Cox proportional hazards regression model was applied to identify predictive factors for DFS.

**Results:**

Of the 50 patients, 42 (84%) were PD-L1 positive. Forty-one cases were *BRAF* mutation-positive, and 38 of them (92.68%) showed PD-L1 positivity, indicating a significant association between the two (*p* = 0.003). No significant correlations were found between PD-L1 and other major indicators, including CD8^+^ T cells (*p* = 0.247), FoxP3^+^ cells (*p* = 0.702), and pathological subtype (*p* = 0.667). In exploratory survival analysis, patients with high CD8^+^ T cell infiltration exhibited a tendency of prolonged DFS compared with those with low infiltration (HR = 0.112, 95%CI: 0.014–0.913, *p* = 0.041).

**Conclusion:**

PD-L1 expression is frequently observed in AM with *BRAF^V600E^* mutation, whereas no significant correlation is identified between PD-L1 and the clinicopathological parameters associated with AM. Within the limitation of relatively short follow-up duration, high CD8^+^T cell infiltration was indicated to be potentially correlated with favorable DFS in AM patients, which needs further verification in cohorts with longer follow-up.

## Introduction

Ameloblastoma (AM) ranks among the most prevalent odontogenic neoplasms of the oral and maxillofacial region, accounting for approximately 10% of all maxillary tumors and with an annual incidence of 0.92 cases per million population (1). According to the 2022 World Health Organization (WHO) classification of head and neck tumors (2), AM can be classified into three subtypes with distinct clinical manifestations: conventional, unicystic, and peripheral types. Among them, conventional AM is the most common subtype, with plexiform and follicular patterns as the main histological features. Although generally benign, this tumor exhibits local invasiveness and a high recurrence rate, and may progress to malignant ameloblastic carcinoma (AC) in advanced stages, resulting in severe maxillofacial deformities and functional impairments. Unicystic ameloblastoma (UAM) is considered a less aggressive variant of AM and is amenable to more conservative treatment. Despite differences in therapeutic approaches, a high prevalence of *BRAF^V600E^* mutation (33%–92%) has been detected in mandibular AM of all subtypes (3–5).

In recent years, the identification of the *BRAF^V600E^* mutation in AM has paved new avenues for targeted therapy (6). The high incidence of this mutation in AM suggests that the MAPK signaling pathway plays a pivotal role in tumor pathogenesis and progression, thereby supporting the potential efficacy of *BRAF* inhibitors in this patient cohort (7). Nevertheless, as observed in other malignancies, single-agent *BRAF*-targeted therapy is often constrained by the emergence of drug resistance and may be accompanied by severe adverse events, which has spurred growing interest in combined immunotherapy regimens (8). Through comprehensive evaluation of Programmed cell death ligand 1 (PD-L1) expression and tumor-infiltrating lymphocytes (TILs) as predictive biomarkers to guide immunotherapeutic decisions is gaining widespread recognition in oncology practice (9).

PD-L1, a key immune checkpoint molecule, facilitates tumor immune evasion by binding to the Programmed death 1 (PD-1) receptor on T cell surfaces and dampening anti-tumor immune responses. Inhibition of the PD-L1/PD-1 interaction using specific antibodies has demonstrated favorable antitumor efficacy across various head and neck tumors (10). However, studies focusing on PD-L1 AM remain limited. Only two investigations have reported PD-L1 positivity rates ranging from 50% to 75% in AM (11, 12). One of these studies further revealed that PD-L1 modulates recurrence and DFS in AM by regulating cell stemness, proliferation, and p-EMT (13). Nevertheless, whether PD-L1 expression is associated with the prevalent *BRAF^V600E^* mutation and whether it influences the immune microenvironment of AM have not yet been fully elucidated.

Immune cells within the tumor microenvironment (TME) exert a critical modulatory effect on immunotherapy responses (14). CD8^+^ cytotoxic T lymphocytes (CTLs) represent the primary effector cells of anti-tumor immunity, yet their activity can be abrogated via the PD-1/PD-L1 axis, leading to T cell exhaustion (15, 16). In parallel, FoxP3^+^ T cells foster an immunosuppressive TME by inhibiting the CD8^+^ T cells and the production of interferon-γ (IFN-γ) (17), and are frequently linked to unfavorable prognoses in a variety of solid tumors (18). Therefore, a comprehensive understanding of the interplay between PD-L1 expression and immune cell infiltration within the TME is essential for delineating the immunobiological properties of AM and informing future therapeutic strategies.

In summary, the present study aimed to evaluate the correlation between PD-L1 expression and the molecular characteristics as well as biological behaviors of AM, and to explore its prognostic evaluation value in patients with different histological subtypes of AM.

## Materials and methods

### Patient cohort

This retrospective observational cohort study enrolled 50 patients with a pathological diagnosis of AM between January 2019 and January 2026 at the First Affiliated Hospital of Bengbu Medical College, all with complete formalin-fixed paraffin-embedded (FFPE) tissue specimens and full clinical data. Clinical and histological subtypes were independently reviewed by two senior pathologists (WQ and CZN) according to the 2022 WHO Classification of Head and Neck Tumors: Pathology and Genetics. Histological subtypes were classified as follicular, plexiform, granular cell, and basal cell types. Discrepant cases were further evaluated by a third senior pathologist (WDN), and a final consensus was achieved after discussion. Recurrence or disease progression was confirmed by maxillofacial CBCT obtained during regular follow-up. Exclusion criteria were as follows: (1) decalcified FFPE tissue; (2) tumor cell proportion less than 30% observed on HE-stained sections; (3) incomplete clinical and pathological data; (4) presence of autoimmune diseases, HIV infection, or syphilis. This study was approved by the Ethics Committee of the First Affiliated Hospital of Bengbu Medical College (approval number: [2019]103X01) and conducted in accordance with institutional guidelines, national regulations, and the Declaration of Helsinki. Written informed consent was obtained from all patients or their legal guardians prior to the collection of all experimental and control specimens.

### Immunohistochemical staining

FFPE tumor sections were deparaffinized with xylene, rehydrated through a graded ethanol series (100%–70%), and rinsed with phosphate-buffered saline (PBS). Antigen retrieval was performed using citrate buffer (pH 6.0) with autoclaving for 2–2.5 minutes. Endogenous peroxidase activity was blocked with 3% hydrogen peroxide solution in methanol for 10 minutes. After rinsing with PBS, the edges of the sections were outlined with a hydrophobic barrier pen, and non-specific binding was blocked with normal goat serum for 20 minutes at room temperature. Primary antibodies were added, and the sections were incubated overnight at 4 °C in a humidified and light-protected chamber. The antibodies used were as follows: PD-L1 (clone 22C3, dilution ratio 1:100; Agilent, Singapore), *BRAF^V600E^* (clone RM8, dilution ratio 1:200; Maxim, China), CD8 (clone SP16, ready-to-use; Maxim, China), and FoxP3 (clone 236A, ready-to-use; Maxim, China). Tonsil tissue and PBS served as positive and negative controls, respectively; tumor stromal tissue was used as an internal negative control. On the following day, slides were equilibrated to room temperature, rinsed with PBST (PBS containing 0.1% Tween-20), incubated with horseradish peroxidase (HRP)-conjugated secondary antibody for 30 minutes, and visualized with 3,3’-diaminobenzidine (DAB) chromogen. Finally, slides were counterstained with hematoxylin, dehydrated, cleared, and mounted with coverslips.

### Quantitative microscopic analysis

High-resolution images of the stained sections were acquired using a fully automated digital slide scanner (VS200). Whole-slide imaging (WSI) of the immunohistochemical sections was scanned with the KFSlideOS software (KFBio), and automated counting of positive cells was performed using QuPath 0.60 software.

### *BRAF ^V600E^* expression assessment

Two senior pathologists independently validated *BRAF^V600E^* expression in all specimens. A case was considered positive when >50% of tumor cells exhibited diffuse cytoplasmic staining with minimal stromal background staining. Cases with focal nuclear staining, pericytoplasmic staining, or mixed epithelial-stromal staining were classified as negative.

### PD-L1 expression assessment

For PD-L1 staining scoring, tissue regions of whole-slide digital PD-L1 sections were first delineated using QuPath software and classified into tumor cells, immune cells, and stromal cells. Automated quantitative and standardized evaluation of PD-L1 membrane staining was then performed. Positive staining was defined as partial or complete membrane staining distinct from cytoplasmic staining. We further quantified PD-L1 expression using the combined positive score (CPS), calculated as:CPS = (number of PD-L1-positive tumor cells + number of positive immune cells)/total number of viable tumor cells×100.With reference to the PD-L1 CPS stratification for head and neck tumors established by Uppaluri et al. (19), we categorized patients potentially eligible for anti-PD-L1 therapy into three groups: CPS<1, 1≤CPS<10, and CPS≥10. A CPS<1 was defined as negative, whereas CPS≥1 was defined as positive. Higher CPS values indicated a higher proportion of PD-L1-expressing cells.

This quantitative scoring strategy has been validated for reliability in several studies of head and neck squamous cell carcinoma (20, 21).

### Infiltration analysis of CD8^+^ and FOXP3^+^ T cells

CD8^+^ and FOXP3^+^ T cell infiltration was quantified at 400× magnification. After demarcation of tumor regions by a pathologist, three representative high-power fields (HPFs; approximately 0.25 mm² per field) were selected from both the tumor parenchyma and stroma for each case, and the density of CD8^+^ or FOXP3^+^ positive cells was calculated as the number of positive cells per mm². CD8^+^ positivity was defined as distinct, complete circumferential membrane staining, whereas FOXP3^+^ positivity was defined as nuclear staining. Cases were divided into high-infiltration or low-infiltration groups based on the median value of positive cell counts. Counting was performed by two independent observers; a review was conducted if the inter-observer Spearman correlation coefficient was <0.85. Tonsil tissue was used as a positive control.

### PCR and Sanger sequencing

Considering that DNA in long-stored FFPE samples may be fragmented, which could compromise PCR and Sanger sequencing results, 29 of the 50 FFPE specimens collected within the recent two years were selected for molecular analysis. DNA was extracted from five unstained, non-decalcified, 5-μm-thick FFPE sections of each sample using the FastPure FFPE DNA Isolation Kit (Vazyme, Nanjing, China) in strict accordance with the manufacturer’s instructions. The PCR reaction mixture contained Phanta Max Master Mix (2×) (P515-01, Vazyme, Nanjing, China), forward (5′-TCA TAA TGC TTG CTC TGA TAG GA-3′) and reverse (5′-GGC CAA AAA TTT AAT CAG TGG A-3′) primers (10 μM), nuclease-free water, and approximately 40 ng of purified genomic DNA. PCR amplification was performed under the following conditions: 95 °C for 5 min; 35 cycles of 95 °C for 30 sec, 60 °C for 1 min, and 72 °C for 1 min; with a final extension at 72 °C for 5 min. Amplified products were analyzed by electrophoresis on 2% agarose gels following staining with FD Green Buffer. Sanger sequencing was performed using an Applied Biosystems 3730xl DNA Analyzer (Baseclear, Leiden, the Netherlands).

### Statistical analysis

Statistical analyses were performed using SPSS 27 software (IBM Corporation, Armonk, NY, USA). The Shapiro-Wilk test was used to assess the normality of continuous variables. Descriptive statistics were applied to summarize the demographic and clinicopathological characteristics of the patients. Continuous variables were described as mean ± standard deviation (SD) for normally distributed data or median (interquartile range, IQR) for non-normally distributed data; Chi-square or Fisher’s exact test was used for comparisons between categorical variables, as appropriate, to examine the associations between PD-L1 expression and various clinicopathological features(Fisher’s exact test was used when the theoretical frequency was <5), and comparisons of non-parametric data between two groups were performed using the Mann-Whitney U test. Disease-free survival (DFS), defined as the time from treatment to disease recurrence or progression (assessed by MRI/CT follow-up every 3–4 months), was estimated using the Kaplan-Meier method, and differences in survival curves between groups were compared using the log-rank test. Univariate Cox proportional hazards regression analysis was performed to calculate hazard ratios (HR) and 95% confidence intervals (CI) for each parameter, in order to evaluate their associations with disease-free survival (DFS). A two-tailed P<0.05 was considered statistically significant.

## Results

### Clinicopathological characteristics

This study enrolled 50 patients diagnosed with AM between January 2019 and January 2026 at Bengbu Medical University. Of these, 32 were male (64%) with a median age of 30 years (range 7–85 years), and 18 were female (36%) with a median age of 39.5 years (range 16–71 years). A total of 44 cases (88%) were mandibular AMs, and 6 were maxillary AMs. According to clinical classification, 39 cases (78%) were multicystic, 8 (16%) were unicystic, and 3 (6%) were extraosseous/peripheral type.

Follicular and plexiform patterns were the predominant histopathological subtypes, accounting for 21 (42%) and 22 cases (44%), respectively. In addition, there were 5 cases (10%) of acanthomatous type, and 1 case each (2%) of granular cell type and basal cell type. Radical treatment (mandibular segmental resection) was performed in 24 patients (48%), while conservative treatment (curettage/enucleation or marsupialization) was administered in 26 patients (52%). The median and maximum follow-up periods were 17 months and 64.5 months, respectively (**Table 1**).

**Table 1:** Clinicopathological characteristics of patients with AM.

Clinicopathological	n	%
Sex		
Female	18	36
Male	32	64
Age (years)		
<40	26	52
≥40	24	48
Size (cm)		
<4	14	28
≥4	36	72
Anatomic location		
Mandible	44	88
Maxilla	6	12
Recurrence status		
Primary	42	84
Recurrent	8	16
Surgical approach		
Conservative surgery	26	52
Radical surgery	24	48
Clinical subtype		
Conventional	39	78
Unicystic	8	16
Peripheral	3	6
Histological subtypes		
Follicular	21	42
Plexiform	22	44
Acanthomatous	5	10
Granular	1	2
Basaloid	1	2
BRAFV600E mutation status		
mutant	41	82
wild-type	9	18

### Correlation between PD-L1 expression and *BRAF^V600E^* mutation status

**Figure 1:**
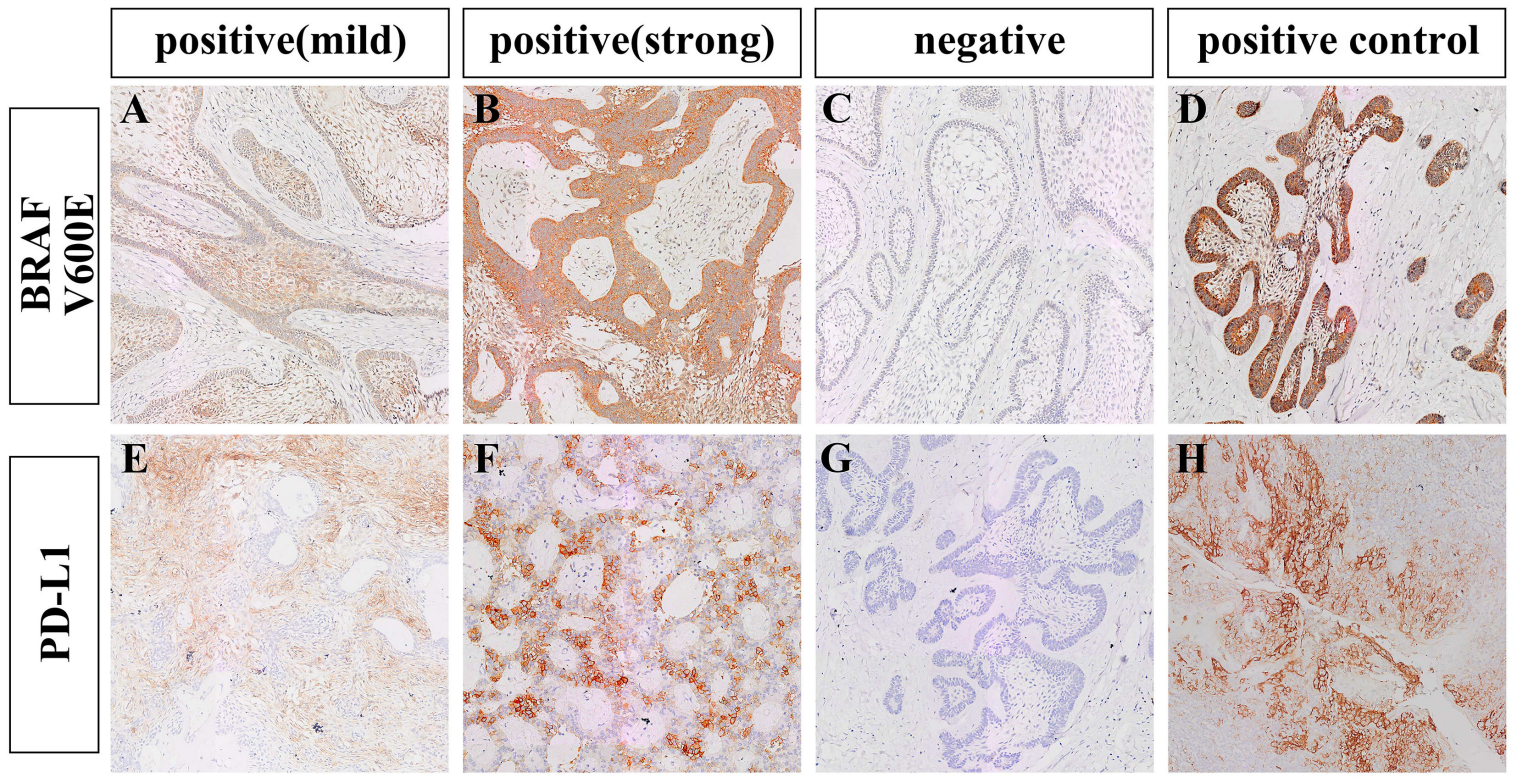
Expression of immune checkpoint molecules and BRAF V600E mutation status in AM. Representative IHC staining images showing BRAF V600E mutations **(A–C)**, positive controls for BRAF **(D)**, PD-L1 expression (CPS≥1) **(E–G)**, and positive controls for PD-L1 **(H)**, n = 50.

**Figure 2:**
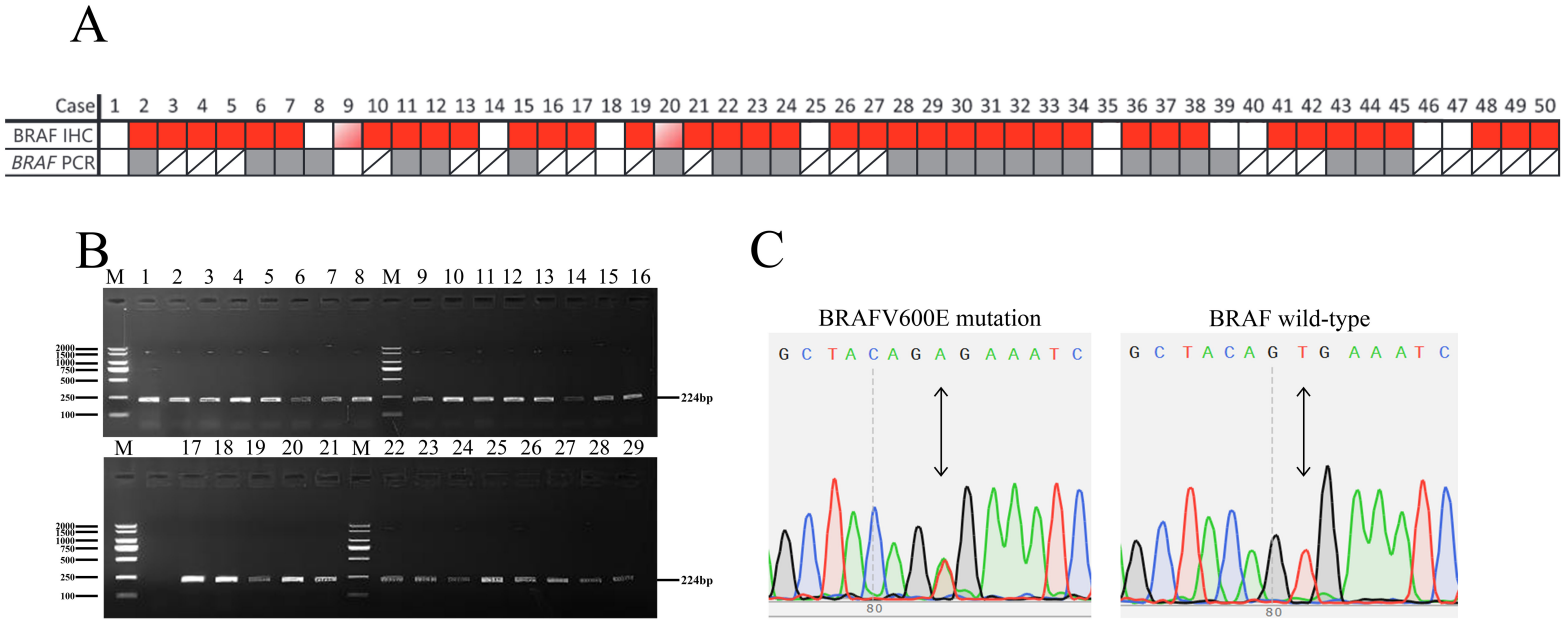
**(A)** Concordance between *BRAF^V600E^* mutation status determined by immunohistochemistry and PCR−Sanger sequencing (positive cases are shown in light red and gray, negative cases in white); **(B)** Agarose gel electrophoresis of PCR amplification products; **(C)** Representative sequencing chromatogram showing the BRAF c.1799T>A mutation.

*BRAF* IHC results showed that 41 of 50 AMs (82%) were positive for the *BRAF^V600E^* mutation. Specifically, the positive rate of *BRAF^V600E^* mutation was 76.92% (30/39) in polycystic AM, 100% (8/8) and 66.67% (2/3) in unicystic and peripheral AM, respectively. Further classification according to histological subtypes revealed that the positive rate of *BRAF^V600E^* mutation was 76.19% (16/21) in follicular type, 86.36% (19/22) in plexiform type, and 100% (5/5) in keratinizing type. However, no correlation was found between *BRAF^V600E^* mutation and clinical subtypes (p=0.479) or histological subtypes (p=0.446). The positive rate of *BRAF^V600E^* mutation in mandibular AM was 84.1% (37/44) (**Figure 1**). In 4% (2/50) of cases, IHC staining was ambiguous, and consensus could not be reached between the two pathologists. PCR and Sanger sequencing were subsequently performed on 29 recent cases with sufficient tumor cells and no decalcification; mutation analysis results are presented in **Figure 2**. The results were consistent with IHC in 25 cases (86.21%) and discordant in 2 cases (6.8%). The consistency between the IHC results of *BRAF^V600E^* mutation and Sanger sequencing results reached 86.21%. Based on Sanger sequencing, the *BRAF^V600E^* mutation status of the 2 IHC−ambiguous cases was finally determined.

Among the 50 patients, 42 (84%) exhibited positive PD-L1 membrane expression with a CPS ≥ 1, while 8 (16%) were PD-L1 negative. Of the 42 PD-L1–positive patients, 38 (90.5%) harbored the *BRAF^V600E^* mutation, compared with 3 of 8 PD-L1–negative patients (37.5%). The *BRAF^V600E^* mutation was significantly associated with increased PD-L1 expression (*p* = 0.003) (**Table 2**).

**Table 2:** The correlation between PD-L1 expression and clinicopathological characteristics.

Clinicopathological Features	PD-L1 positive (n=42)	PD-L1 negative (n=8)	*p*
Sex			0.436
Male	28	4	
Female	14	4	
Age (years)			0.456
<40	23	3	
≥40	19	5	
Tumor size (cm)			1.000
<4	12	2	
≥4	30	6	
Anatomic location			0.242
Mandible	38	6	
Maxilla	4	2	
Recurrence status			0.324
Primary	34	8	
Recurrent	8	0	
Surgical approach			0.261
Conservative surgery	21	6	
Radical surgery	21	2	
BRAFV600E mutation status			0.003*
mutant	38	3	
wild-type	4	5	
Clinical subtype			0.686
Conventional	33	6	
Unicystic	7	1	
Peripheral	2	1	
Histological subtypes			0.667
Follicular	16	5	
Plexiform	19	3	
Acanthomatous	5	0	
Granular	1	0	
Basaloid	1	0	
CD8^+^T cell infiltration			0.247
Positive	19	6	
Negative	23	2	
FOXP3^+^T cell expression			0.702
Positive	22	3	
Negative	20	5	

* Statistical significance at *p* < 0.05.

### Correlation between PD-L1 expression and CD8^+^/FOXP3^+^ T cell infiltration

**Figure 3:**
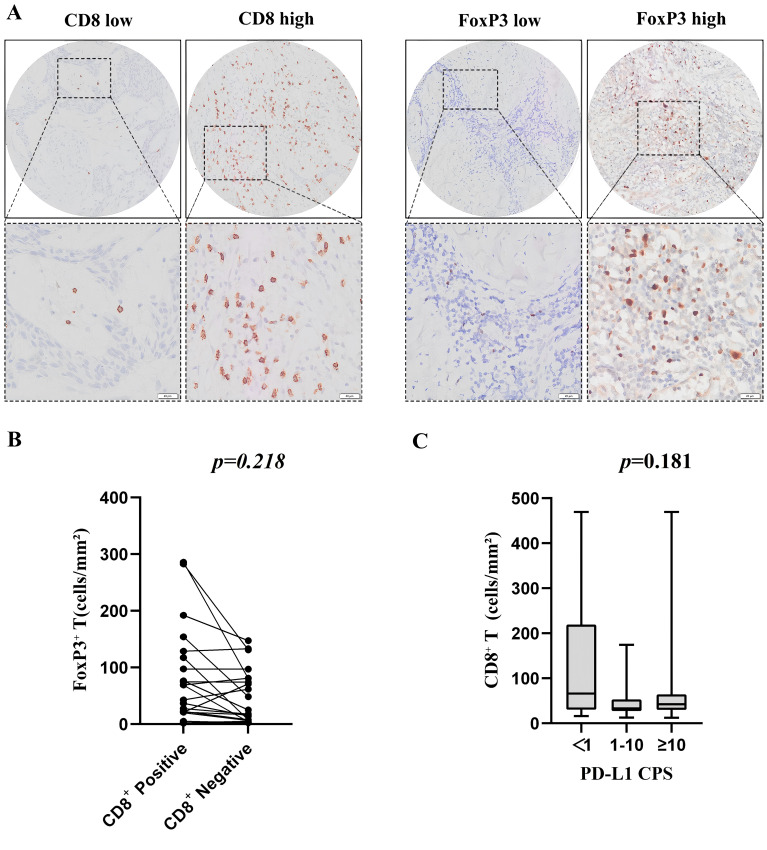
**(A)** Representative IHC staining of tumor-infiltrating lymphocytes in AM (n = 50). Scale bar: 20 µm. **(B)** Comparison of FoxP3^+^ T cell continuous variables between CD8^+^ T cell high- and low-infiltration groups (*p* = 0.218). **(C)** Differences in CD8^+^ T cell infiltration evaluated across PD-L1 CPS subgroups (*p* = 0.181). AM, ameloblastoma; TILs, tumor-infiltrating lymphocytes.

Using the median CD8^+^ T-cell count (42.095 cells/mm²) as the cutoff value, 25 patients (50%) were classified as CD8^+^ T-cell positive (**Figure 3A**). Of these 25 CD8^+^ T-cell–positive patients, 19 (76%) were PD-L1 positive and 6(24%) were PD-L1 negative. However, no significant association was observed between CD8^+^ T-cell infiltration and PD-L1 expression (*p* = 0.247) (**Table 2**).

FoxP3^+^ T cells were also stratified using the median value as the cutoff (**Figure 3A**). Among the 42 PD-L1–positive patients, 22(52.38%) were FoxP3^+^ T-cell positive (*p* = 0.702). Correlation analysis between continuous FoxP3^+^ and CD8^+^ T-cell variables showed no significant association (*p* = 0.218) (**Figure 3B**).

Further analysis was performed using a three-tiered PD-L1 CPS classification: CPS<1, 1≤CPS<10, and CPS≥10. Again, no significant correlation was found between PD-L1 expression and CD8^+^ T-cell infiltration (*p* = 0.181). Notably, the median CD8^+^ T-cell infiltration density was relatively lower in the PD-L1 CPS 1–10 subgroup (32.925 cells/mm²) (**Figure 3C**).

### Disease‐free survival analysis

To evaluate the prognostic impacts of PD-L1 expression, *BRAF^V600E^* mutation, and immune microenvironment markers, univariate Cox regression analysis was performed on 50 patients with complete tumor samples. The median follow-up time was 12 months (range: 3–67 months), during which 8 patients (16%) developed local recurrence. Univariate analysis revealed that CD8^+^ T cell infiltration (HR = 0.112, 95% CI: 0.014–0.913, *p* = 0.041) was a significant protective factor against tumor recurrence or progression (**Table 3**), with high CD8^+^ T cell infiltration associated with significantly improved DFS.

**Table 3:** Univariate analysis of clinicopathological factors associated with DFS.

Univariate analyses (Cox)					
Clinicopathological Features	n	Events (recurrences)	HR	95% CI	*p*
Sex			0.645	0.150-2.765	0.555
Male	18	5			
Female	32	3			
Age (years)			0.128	0.016-1.038	0.054
<40	26	7			
≥40	24	1			
Tumor size (cm)			0.901	0.180-4.510	0.899
<4	14	2			
≥4	36	6			
Anatomic location			0.208	0.040-1.084	0.062
Mandible	44	6			
Maxilla	6	2			
Surgical approach			0.702	0.167-2.946	0.628
Conservative surgery	26	5			
Radical surgery	24	3			
BRAFV600E mutation			0.036	.000-90.355	0.406
mutant	41	8			
wild-type	9	0			
Clinical subtype			1.827	0.686-4.870	0.228
Conventional	39	5			
Unicystic	8	2			
Peripheral	3	1			
Histological subtypes			0.977	0.409-2.332	0.958
Follicular	21	2			
Plexiform	22	6			
Acanthomatous	5	0			
Granular	1	0			
Basaloid	1	0			
CD8^+^T cell infiltration			0.112	0.014-0.913	0.041*
Positive	25	1			
Negative	25	7			
FoxP3^+^T cell expression			2.261	0.518-9.873	0.278
Positive	25	5			
Negative	25	3			
PD-L1 expression			23.932	0.001-6183	0.540
Positive	42	8			
Negative	8	0			

* Statistical significance at *p* < 0.0.5.

**Figure 4:**
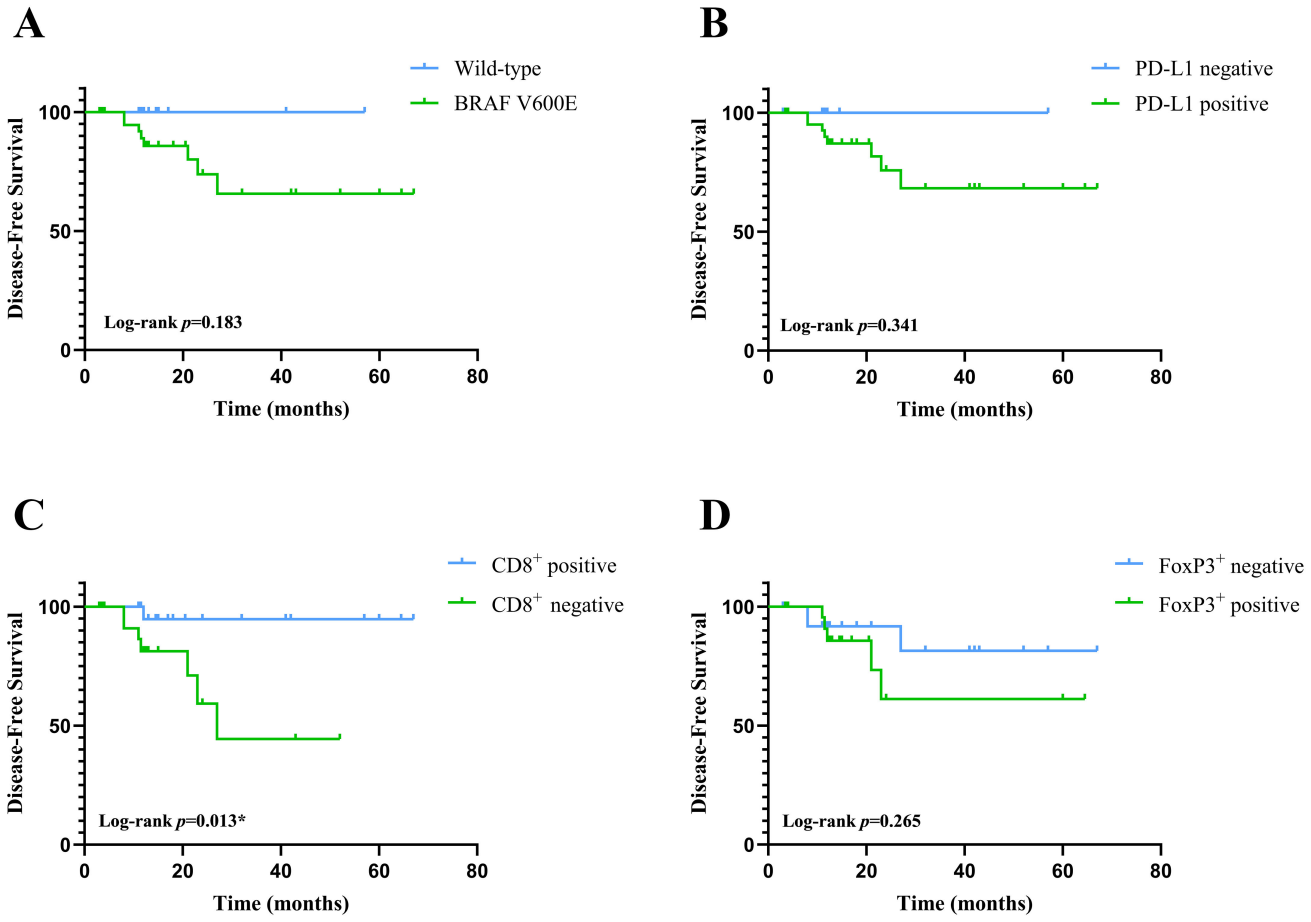
Kaplan–Meier survival curves. **(A)** *BRAF^V600E^* mutant versus wild-type; **(B)** Negative versus positive PD-L1 expression; **(C)** High versus low CD8^+^ T cell infiltration; **(D)** High versus low FoxP3^+^ T cell infiltration.

In contrast, no statistically significant differences in DFS were observed for *BRAF^V600E^* mutation status (HR = 0.036, 95% CI: 0.000–90.355, *p* = 0.406), PD-L1 expression (HR = 23.932, 95% CI: 0.001–6183.000, *p* = 0.540), or FoxP3^+^ T cell infiltration (HR = 2.261, 95% CI: 0.518–9.873, *p* = 0.278) (**Figure 4**).

## Discussion

In the present study, we performed IHC profiling of PD-L1 expression and CD8^+^ and FoxP3^+^ T-cell infiltration in AM, and analyzed the common *BRAF^V600E^* mutation in AM using IHC as well as PCR and Sanger sequencing. Several recent studies on AM have reported elevated PD-L1 expression, which influences tumor recurrence and growth (11, 13). This raises the question of whether PD-L1 expression is associated with key clinicopathological features and prognosis in AM. Given the encouraging efficacy of targeted therapies against *BRAF^V600E^* mutation (7), there is growing interest in whether PD-L1 may serve as a predictive biomarker for immunotherapy, with the potential to combine targeted and immune-based strategies to reduce drug resistance and treatment-related toxicity.

A previous study investigating PD-L1 expression in AM demonstrated that PD-L1 promotes tumor growth and recurrence (13), which is inconsistent with the findings of the present study. In our research, PD-L1 expression showed no significant correlation with clinicopathological factors, such as tumor size, location, clinicopathological classification, and recurrence status. One possible reason is that the optimal threshold for PD-L1 expression in AM has not yet been validated. Currently, only a limited number of thresholds (including 1% and 1–49%) are used to determine PD-L1 positivity; however, the overall scores are relatively low while showing a predominantly positive trend (11, 12). Therefore, based on previous studies on PD-L1 immune scoring in head and neck tumors, the present study defined the PD-L1 threshold as CPS = 1, and further stratified PD-L1 expression into three grades according to the specific CPS scores obtained: <1, 1–10, and >10 (22–24). In addition, to reduce experimental errors, consistent with the previous two studies, we used the PD-L1 monoclonal antibody clone 22C3 for IHC staining. Another possible factor contributing to the lack of correlation between PD-L1 expression and clinicopathological subtypes is the limited sample size enrolled in this study. A total of 43 cases (86%) were plexiform or follicular AM, while only 5 cases, 1 case, and 1 case were acanthomatous, granular cell, and basal cell AM, respectively. Since AM of different pathological subtypes exhibit distinct clinical characteristics, it is necessary to increase the sample size of rare subtypes in future studies.

In addition, this study found a significant positive correlation between *BRAF^V600E^* mutation expression and PD-L1 expression in AM (*p* = 0.003). Combined with the molecular characteristics of AM, the *BRAF^V600E^* mutation primarily drives the persistent activation of the MAPK/ERK signaling pathway. A study by Zhang et al. demonstrated that PD-L1 expression is regulated by the PI3K–AKT–mTOR pathway, which influences the proliferation of AM cells and partial epithelial-mesenchymal transition (EMT), thereby promoting tumor growth and recurrence. We speculate that PD-L1 expression may be regulated by upstream signaling networks at multiple levels. Meanwhile, a study by Antonino et al. indicated that the PAM pathway often cross-regulates with the Ras/ERK pathway, and mutations in PI3K and *BRAF* genes frequently co-occur in various cancer types (25). Based on the significant correlation between *BRAF^V600E^* mutation and PD-L1 expression observed in this study, a reasonable explanation is that there may be crosstalk and synergistic carcinogenic effects between the MAPK pathway and the PI3K-AKT-mTOR pathway.

CD8^+^ T cells are the core effector cells of the anti-tumor immune response, and their infiltration level is closely associated with the prognosis of various solid tumors (26, 27). In the present study, no significant correlations were observed between PD-L1 expression and CD8^+^ T cell infiltration, nor between FoxP3^+^ regulatory T cells and CD8^+^ T cells. However, the DFS was significantly prolonged in the high CD8^+^ T cell infiltration group (HR = 0.112, 95% CI: 0.014–0.913, *p* = 0.041), suggesting that CD8^+^ T cells may act as an independent prognostic factor without relying on immune checkpoint expression or immunosuppressive cell infiltration. This phenomenon may be related to the complexity and heterogeneity of the tumor immune microenvironment. PD-L1 expression can be regulated by multiple mechanisms, including intrinsic tumor oncogenic signals and microenvironmental stimuli, and thus may not have a linear correlation with CD8^+^ T cell density. Similarly, the impact of FoxP3^+^ regulatory T cells on anti-tumor immunity may depend on their functional status and spatial distribution rather than their absolute quantity. A previous study confirmed the presence of CD8^+^ T cells in the tumor stromal region of AM (6). Therefore, the better DFS in the high CD8^+^ T cell infiltration group indicates that CD8^+^ T cells play a positive role in the tumor immune microenvironment (28). We speculate that PD-L1-targeted inhibitors combined with other drugs may be beneficial for prolonging treatment response and delaying the accumulation of drug resistance.

None of the clinical, mutation, histological, or immunohistochemical characteristics included in this study were associated with the DFS of AM, indicating their limited value in predicting prognosis. However, multiple studies have shown that the surgical approach is a significant factor associated with DFS (29–31). This may be attributed to the fact that the hospital where the authors are affiliated is a regional central hospital, where a certain number of patients were admitted with multiple recurrences. Therefore, radical treatment is more commonly adopted in this hospital. Given the inherent biological nature of ameloblastoma with indolent growth and a high tendency of late recurrence usually occurring more than 5 years after surgery, limited follow-up duration may lead to under-detection of recurrence events and introduce bias into DFS analysis. Although only 8 out of 50 patients experienced postoperative recurrence, it must be emphasized that the treatment decision for AM should not rely solely on the tumor’s recurrence potential. This is because radical surgery is associated with an increased incidence of postoperative complications and sequelae, which significantly affect patients’ quality of life.

This study has certain limitations: First, the small sample size and single-center retrospective design may introduce selection bias. The sample sizes of some pathological subgroups (e.g., AM with granular cell type and basal cell type) were too small, and only 8 cases of postoperative recurrence were included in this cohort, which exerted a certain impact on statistical power. Second, the median follow-up time was 12 months, while the peak recurrence of AM may occur more than 5 years after surgery (6), making the current follow-up duration insufficient to fully evaluate long-term prognosis. Third, Insufficient fresh tissue samples were collected to verify the relationships among immune markers using flow cytometry.

Future research can be further improved in the following aspects: expanding the sample size and conducting multicenter prospective studies, extending the follow-up time to clarify the long-term prognostic value of molecular-immune markers. genomic and/or proteomic datasets incorporating AM patient data, and to conduct in-depth statistical analyses on them; using flow cytometry、spatial transcriptomics or multiplex immunofluorescence technology to accurately characterize the spatial distribution of the AM immune microenvironment and intercellular interactions; verifying the molecular mechanism of PD-L1 expression regulated by the BRAF-MAPK pathway through *in vitro* cell experiments and animal models to provide target validation for combination therapy; conducting clinical exploration of BRAF inhibitors combined with PD-1/PD-L1 inhibitors in patients with recurrent or unresectable AM to systematically evaluate the safety and efficacy of this treatment regimen.

## Data Availability

The raw data supporting the conclusions of this article will be made available by the authors, without undue reservation.
